# On-Demand Indexing for Referential Compression of DNA Sequences

**DOI:** 10.1371/journal.pone.0132460

**Published:** 2015-07-06

**Authors:** Fernando Alves, Vinicius Cogo, Sebastian Wandelt, Ulf Leser, Alysson Bessani

**Affiliations:** 1 LaSIGE, University of Lisbon, Lisbon, Portugal; 2 WBI, Humboldt-Universität zu Berlin, Berlin, Germany; Centro de Investigación y de Estudios Avanzados del IPN, MEXICO

## Abstract

The decreasing costs of genome sequencing is creating a demand for scalable storage and processing tools and techniques to deal with the large amounts of generated data. Referential compression is one of these techniques, in which the similarity between the DNA of organisms of the same or an evolutionary close species is exploited to reduce the storage demands of genome sequences up to 700 times. The general idea is to store in the compressed file only the differences between the to-be-compressed and a well-known reference sequence. In this paper, we propose a method for improving the performance of referential compression by removing the most costly phase of the process, the complete reference indexing. Our approach, called On-Demand Indexing (ODI) compresses human chromosomes five to ten times faster than other state-of-the-art tools (on average), while achieving similar compression ratios.

## Introduction

A genome is the complete set of DNA from an organism, and contains almost all information needed to build it and maintain it alive. In humans, the entire genome sequence is comprised of more than 3 billion DNA base pairs (bp), which are enclosed in all cells of a body. The genome typically is stored as a 3GB text-based file (one byte per bp).

The cost of genome sequencing has decreased exponentially in the last few years [1, 2], and the number of sequenced and stored genomes is increasing at a similar pace [3, 4]. Until recently, sequencing a human genome costed several thousand US dollars. However, the impressive mark of $1,000 for the sequencing of a whole human genome was achieved in the beginning of 2014 by *Illumina Inc.* with the *HiSeq X Ten* platform [5, 6]. Sequencing cost is expected to continue falling in the following years, which creates ample opportunities for biomedical research in general and in particular for personalized medicine. This trend will yield an strong increase in the amount of generated data.

In computer science, compression is a process for reducing the size of data sets for efficient data transfer and storage. Compressing genomes is an important step in many bioinformatics workflows since it attenuates the opening gap between sequencing throughput and storage technologies [7]. However, traditional compression algorithms (e.g., ZIP) are inefficient when working with genomic sequences [8]. Developers of genome handling systems have created compression tools specifically for genomic data, but typically with high latency and resource costs [9].

Referential compression is the state-of-the-art approach for highly similar genomes (e.g., humans have at least 99.5% of genetic similarity), since it generates an output file containing only the differences between two input sequences (a to-be-compressed and a reference sequence) [10]. A common practice in the current tools implementing this approach is to index the entire reference genome before starting the compression. This process ensures optimal compression ratios, as it guarantees that always the best (i.e., longest) match in the reference can be found. However, it is also an expensive step [11]. This cost typically is not taken into account when compressing genomes, assuming that it is amortized by compressing several files at once. But this is not the only, and possible not even the most common, way of using compression tools. Instead, we observed that very often only single genomes are compressed. In such cases, indexing the reference or at least loading a previously generated index must be considered when comparing the runtime of algorithms.

In this paper, we propose the *On-Demand Indexing (ODI)* approach for referential compression of highly similar sequences. It removes the need for indexing the entire reference genome before starting the compression algorithm, which translates in benefits as those from the following two points of view:

- From a *practical* perspective, referential compression is typically employed separately for each individual genome. In this case, one either needs to re-index the entire reference sequence (as is done in all compression tools we are aware of) or to load an “index file” from the disk to a searchable data structure in main memory. In both cases, the time spent in these tasks is substantially larger than the time required to compress non-trivial sequences. Our approach entirely avoids these two steps and thus compresses genomes much faster than traditional tools, while reaching competitive compression ratios, decompression speed, and memory footprint.
- From a *conceptual* perspective, ODI introduces a new kind of design in which having the entire reference index is not a precondition for compression. Instead, small-segment indexes are built only when direct sequence matching fails. The consequence is the removal of a costly first step, dramatically decreasing the time required for running the compression tool, the amount of memory used, and the eventual need for maintaining index files.

The remainder of this paper is organized as follows: we review the most important aspects about referential compression in Section *Referential Compression*. The on-demand indexing approach is detailed in Section *On-Demand Reference Indexing*. Section *Implementation Details* describes the implementation details from our tool, called JDNA. In Section *Results*, we discuss the performance of JDNA and compare it to FRESCO. We conclude the paper in Section *Conclusions*.

## Methods

In this section we present an introduction to referential compression of DNA files, our new algorithm, and some details of our implementation of the mentioned ODI algorithm.

### Referential Compression

The main concept of referential compression for DNA sequences is, given a to-be-compressed sequence and a reference, writing an output file containing only the differences between the two input sequences [10]. This approach obtains the best results when organisms have a high genetic similarity (e.g., humans have at least 99.5% of genetic similarity). The higher the similarity, the higher the compression ratio.

Referential compression is the most efficient approach for compressing DNA sequences. Current algorithms implementing it obtain compression ratios of at least 400:1 (i.e., four hundred to one), meaning the compressed data can be 400× smaller than the original. Non-referential approaches achieve ratios of up to 8:1 [9].

Several tools implement referential compression, and some of them are noteworthy, namely: DNAzip [12], RLZ [13], GDC [14] and FRESCO [15]. DNAzip is one of the pioneers in this approach, and the other mentioned solutions optimized and advanced the topic at their launching time. The main differences between them appears in a survey published in 2013 [9]. FRESCO is currently the fastest solution considering only the compression process, which surpasses the other tools by an order of magnitude [15]. However, FRESCO’s measurements do not include the time required to create the index structure (as is the case for all other evaluations of referential genome compression we are aware of). Note that all these tools and our algorithm compress any contiguous sequence obtained from sequencing data as long as a representative reference is available, but do not compress raw reads with quality information attached, i.e., FASTQ format.

**Fig 1:**
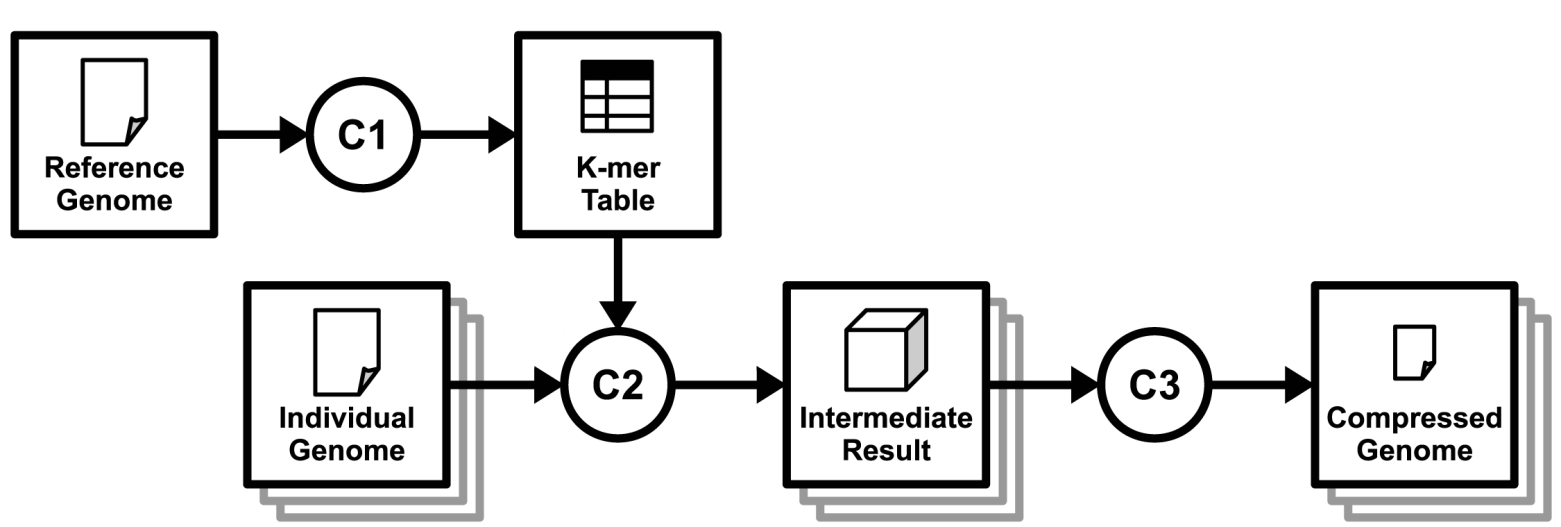
Conceptual model of FRESCO’s execution. (C1) Reference genome indexing. (C2) Compression of the input genome(s), using the indexed reference. (C3) Encoding of the preliminary results to produce the final file.

From this point on, we use only FRESCO as the baseline to our study since it already was compared to and outperforms all other related tools in terms of execution time, and achieves competitive compression ratios [15]. FRESCO is a lossless referential compression library for DNA sequences, stored in RAW or FASTA formats. It is written in C++ and is open source [15]. Compressing a genome in FRESCO requires three steps (see Fig 1): (C1) indexing, (C2) compression itself and (C3) encoding.

#### (C1) Indexing

FRESCO uses a K-mer table [16] to index the complete reference genome. A K-mer is each motif (i.e., a small segment) of length *K* observed in a DNA sequence; the order of a K-mer is defined by its word size. It resembles a hash table, with the difference that it stores a list of values per key. The hash of each segment with size *K* from the reference sequence is calculated and used as a key, and the position where the segment was found is stored in the list of values indexed by this hash. The resulting data structure contains all segments of size *K* from the reference, and is used to find matches between the reference and the to-be-compressed sequences. Indexing the complete reference sequence provides a deterministic search property since lookups in the resulting data structure return if a segment definitely is present or absent in the reference sequence. Once this data structure is ready, it can be reused if maintained in memory or stored in a persistent file that is loaded each time a new compression with that reference is initiated.

#### (C2) Compression

The compression phase uses the referred indexing structure and the to-be-compressed sequence(s). Each K-mer of the to-be-compressed sequence is hashed, and this hash is looked up in the K-mer table. A successful lookup returns a list of indexes where the segment can be found in the reference. The index that produces the longest match is calculated on execution time by extending each match as much as possible through direct base comparison with the reference. A new entry is created in the intermediate result when the match ends. If a match ends in a position *P* of the to-be-compressed sequence, the next lookup on the table will use the segment starting on position *P*+1. If the latest lookup returned an empty list of indexes, then the base pair on position *P*+1 is set as a difference between matches, and a new lookup will be made using the segment of size *K* starting on position *P*+2. This is repeated until a new table lookup provides a match. All base pairs placed between the new and the previous match are also stored in the intermediate result. This method is repeated until the entire to-be-compressed sequence is processed. Each entry, in the intermediate result, is composed of the size of the match and the base pairs differing from the reference until the next found match.

#### (C3) Encoding

The intermediate result, created by the (C2) compression phase, already is delta encoded [17]. Other additional compression algorithms can be applied in the intermediate result (e.g., GZIP [18]). Finally, the compressed result is stored in the output file.

**Fig 2:**
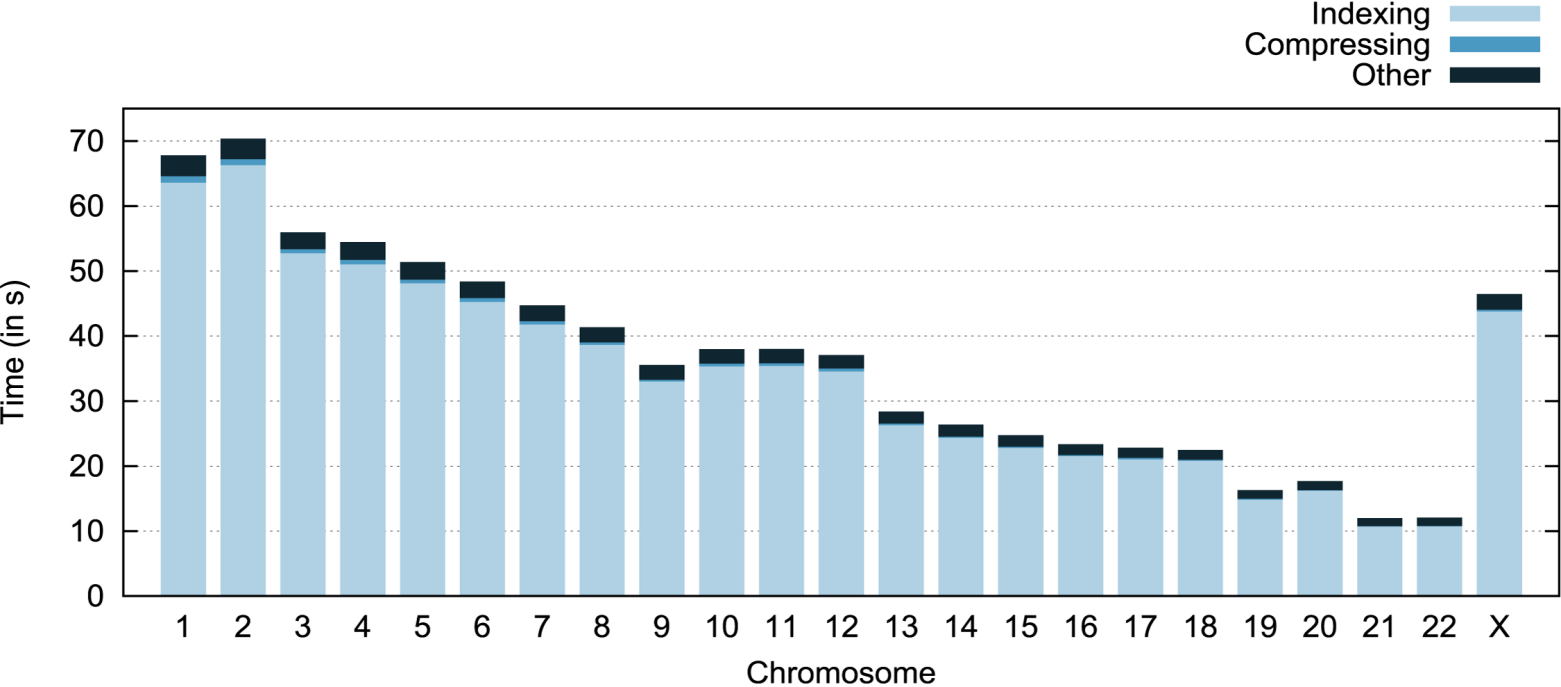
FRESCO’s execution time separated on its internal steps. Time each compression step takes for each chromosome (in proportion).

Fig 2 presents a composite analysis of FRESCO’s execution time for different chromosomes of a human genome (Section *Results* contains a description of our experimental environment). Each bar in this graph comprises the three previously described steps. The “Other” part is composed of several shorter steps, such as computing the encoding, file reading/writing, and variable initialization. This figure shows that most of FRESCO’s execution time is spent in the indexing phase. This phase spends more than 90% of total execution time because the construction of the K-mer table includes adding to this structure every K-mer present in the reference chromosome sequence. For example, the largest human chromosome generates a K-mer table with more than 250 million entries. The compression itself spends only about 1.5% of the execution time, and the remaining execution tasks spend about 4%.

#### (D) Decompression

In the decompression process, the original sequence is rebuilt using the compressed file and the same reference sequence used during the compression. The compressed file is composed of match entries containing the size of matches between the reference and the original to-be-compressed sequences, and base pairs corresponding to the differences between them. The matches and the different base pairs are intercalated. For each entry, FRESCO writes the match and the corresponding base pairs that form the variations between the sequences. FRESCO transcribes a sequence from the reference to the output when writing a match, and writes the different base pairs.

### On-Demand Reference Indexing

In this section, we present the *On-Demand Indexing* (ODI) approach for referential compression algorithms. The following descriptions present our modifications on each step from Fig 1. As demonstrated in the previous section, the indexing step (C1) is responsible for the largest portion of FRESCO’s execution time. ODI avoids indexing the entire reference at the beginning of the program execution. Instead, we propose the use of simple heuristics for sequence comparison to find the matches, and only create a small index when these heuristics provide no results.

#### Compression

The main idea of ODI is to remove the indexing step from the beginning of our workflow execution. This means that the compression step (C2) will not use an auxiliary data structure with the entire reference genome indexed.

During the whole execution, we keep a pointer to the reference (*RP*) and a pointer to the to-be-compressed sequence (*CP*). The former indicates the reference position where we are trying to find matches. The latter is the to-be-compressed position we are trying to match to the reference. Both pointers advance when matches are found. The to-be-compressed sequence is processed searching for matches in the reference until all input has been analyzed. Our compression algorithm executes the following steps iteratively:

1. Search a sequence composed of ambiguous nucleotides (marked as “N”), which are not mapped to the reference. This is a special case verification.
2. Search for a match using the ODI matching algorithm, which will be described in Section *ODI Matching Algorithm*.
3. If the step 2 did not return a match, then we advance *CP* one nucleotide and return to the step 1.
4. If the step 2 provided a match, then we extend the match as much as possible through direct nucleotide comparison.
5. Once the match extension has finished, we write the base pairs between this match and the previous one, write the match itself, and move forward to the step 1. If it is the first match, then we write only the match.

#### ODI Matching Algorithm

Our ODI algorithm searches for matches between the reference and the to-be-compressed sequences, mostly based on simple heuristics. ODI still includes a small index structure, which is instantiated only if the preceding heuristics produce no match. The matching algorithm executes the following steps:

1. **Direct match.** Match segments from the reference and the to-be-compressed sequences directly.
2. **SNP test.** Test if the previous match ended in a Single Nucleotide Polymorphism (SNP).
3. **Brute-force search.** Execute a brute-force search for a match within *δ* base pairs.
4. **Index lookup.** Index Δ base pairs from the reference starting on the current *RP* and perform one table lookup (using the K-mer starting at *CP*), just like FRESCO’s algorithm. If the lookup returns more than one entry, we choose the one which is closer to the *RP*.

**Fig 3:**
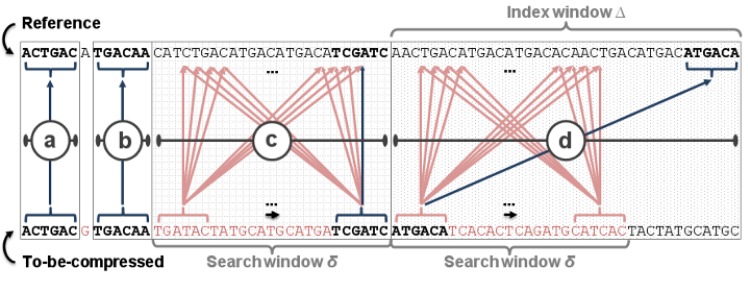
Different matching techniques used in our compression algorithm. ODI uses: (a) Direct match. (b) SNP test. (c) Brute-force search. (d) Index lookup.

Fig 3 illustrates these four steps, which are described in detail in the following paragraphs. Any of these steps immediately returns a match to the compression algorithm (described above) or returns no match.

##### (a) Direct match

This test directly compares the *K* base pairs starting on *RP* and *CP*. If all their nucleotides are equal on each position, then the match is detected and returned.

##### (b) SNP test

This matching algorithm starts by skipping one nucleotide both in the reference and in the to-be-compressed sequence, and performing a direct match (as in the step (a)). If the reference sequence of size *K* starting in the position *RP*+1 is equal to the one from the to-be-compressed sequence starting in the position *CP*+1, then a match is detected and returned. This case detects a SNP, which is the variation type of a considerable large number of genomic variations in human beings.

##### (c) Brute-force search

**Fig 4:**
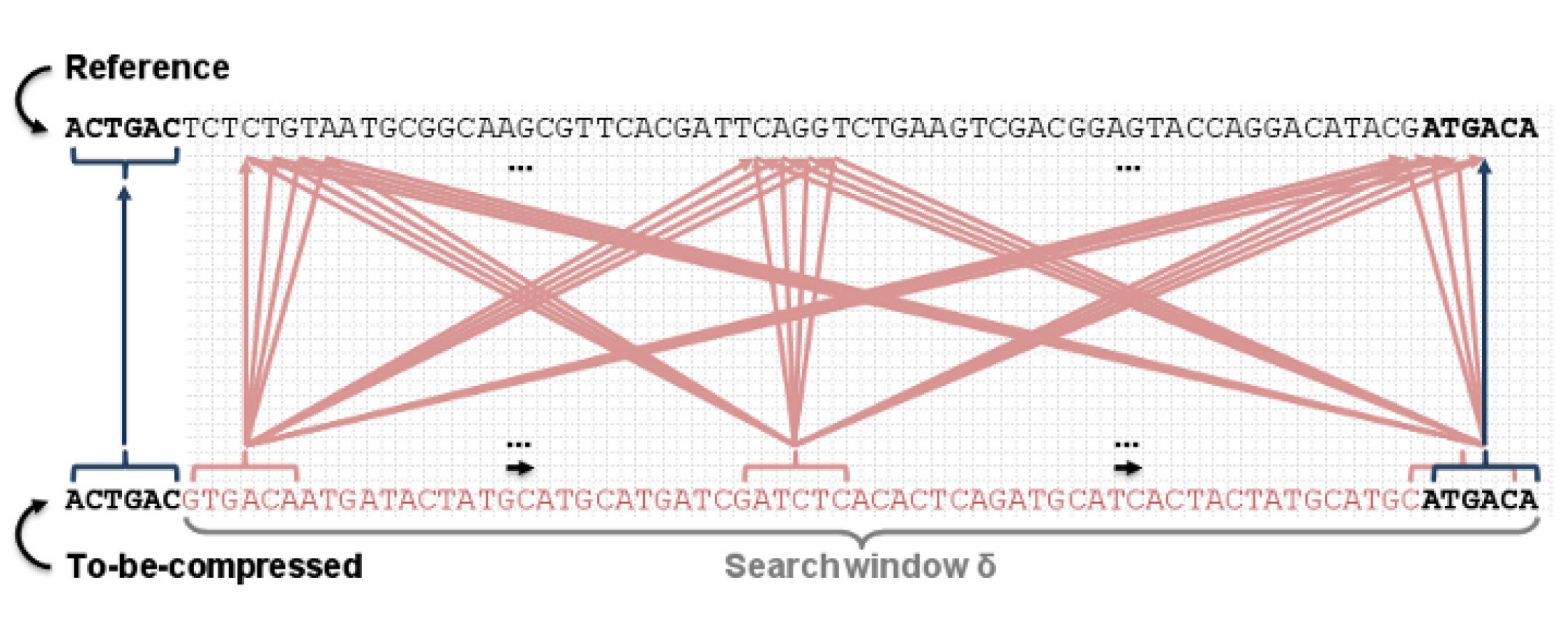
Brute force search. We test every combination of *K* base pairs within the *δ* window for a match.

This test performs a brute-force search within a window of *δ* base pairs when the first two steps failed to find a match. It first tests the to-be-compressed segment of size *K* starting in the position *CP* against all reference segments of size *K* within the *δ* window. Fig 4 shows this matching approach. It covers mutations of up to *δ* − *K* base pairs. The value of *δ* is configurable and should be adjusted to the species of the input sequences.

##### (d) Index lookup

**Fig 5:**
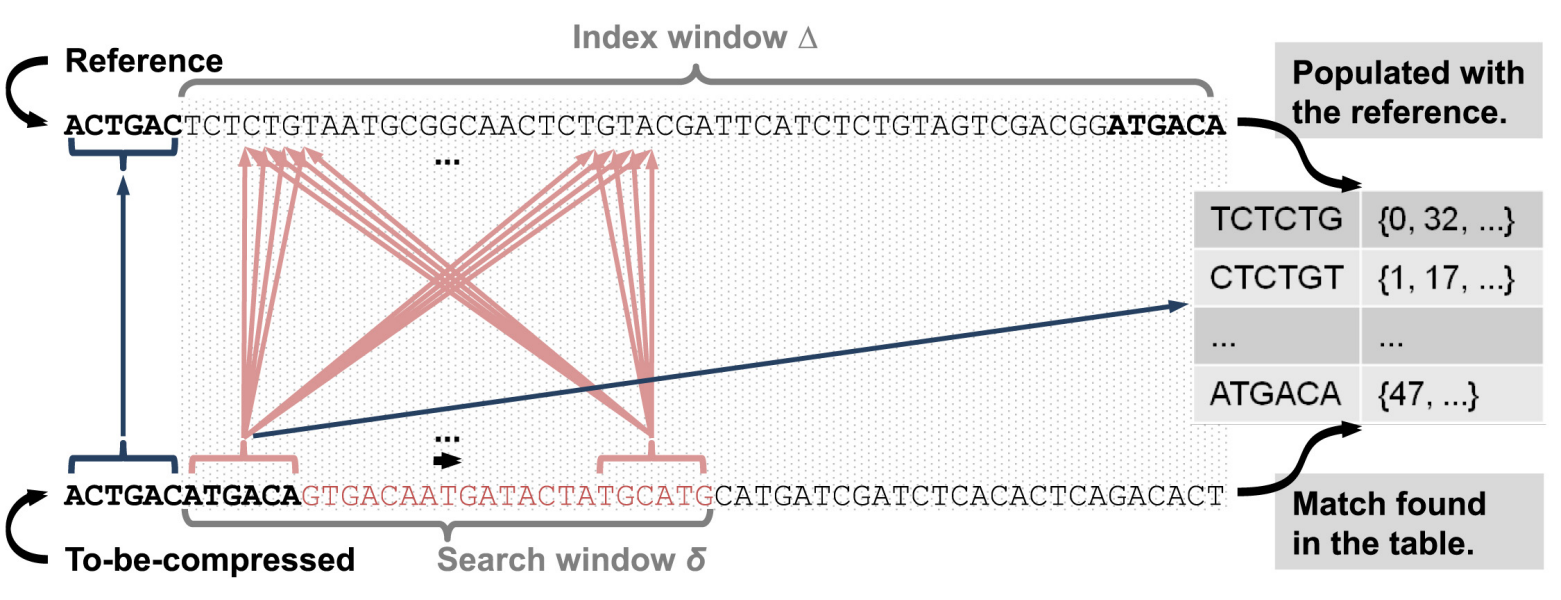
Index lookup. If the search window *δ* does not contain a match, then we index Δ base pairs and execute one table lookup.

Our last method, if all others failed to find a match, indexes a small sequence block from the reference and performs a table lookup. It resembles FRESCO’s algorithm, but it populates the K-mer table with only Δ base pairs instead of the entire reference sequence. Fig 5 shows the point a brute-force search fails, which is the time we start populating the index table and perform a lookup on it. The lookup uses a segment of size *K* from the to-be-compressed sequence. The only difference, compared to FRESCO’s algorithm, is that we select as the best match the value which is closer to *RP*.

The index lookup is instantiated only when the brute-force search do not find a match in the *δ* window. Our experiments suggest using a small Δ value makes our algorithm more time efficient than using large values. However, the Δ window should always include the *δ* one for compression ratio efficiency. If we do not include the *δ* window in the Δ one, then each match entry would include all base pairs from the *δ* window as genomic variations, which would reduce the compression ratio in several cases.

Since our algorithm has no index structure to find genomic variations, we use the described heuristics for this purpose. The direct match is mainly used when JDNA changes compression block (see the next section), or when “N” sequences are detected. SNP matching finds SNPs in the input genome, which is a very frequent genomic variation detected easily. When a SNP test provides no match, an insertion, a substitution, or a deletion is present. Therefore, the brute force and index windows are designed and used to cover any of these genomic variations.

The worst-case time complexity of our ODI matching algorithm is *O*(*δ* ^2^) since the individual steps (direct match, SNP test, brute-force search, index lookup) are executed sequentially and have complexities *O*(1), *O*(1), *O*(*δ* ^2^), *O*(Δ), respectively. Thus, the complexity can be upper-bounded by an appropriate value for *δ*, but this might lower the compression ratio. However, in most cases ODI matches are computed in *O*(1) by one of the first two heuristics since most differences between sequences with high genomic similarity differ in a single nucleotide at time.

#### Advantages and disadvantages of ODI

The main advantage of ODI when compared with other solutions (including FRESCO) is the avoidance of building or loading the index for the entire reference genome before compression. This enables compression to be performed up to one order of magnitude faster, as we show in our results. These fast compression times come at a price, as ODI cannot guarantee to find always the longest match of a segment in the reference, resulting in slightly inferior compression rates. Furthermore, building an index (like FRESCO and others do) pays off if many sequences are to be compressed against the same reference; for such cases, ODI is not the right choice.

### Implementation Details: JDNA

We developed a lossless referential compression tool, called JDNA, using the ODI approach. It is written in Java, and is based on FRESCO’s algorithm with the modifications proposed in Section *On-Demand Reference Indexing*. We present the most important technical details in the present section, and describe the values we used in the configurable options.

In the compression phase, JDNA compresses sequences block by block to reduce the required main memory space. One may configure the block size according with its available resources, and we set 250MB as the default value. Additionally, we implemented a mechanism that reuses the object already created to the K-mer table, instead of instantiating a new K-mer object for the compression of each block.

In the encoding phase, the intermediate result already comes delta encoded. We use Huffman Encoding [19] to increase the efficiency of delta encoding. For example, our Huffman encoding has a special case for SNPs since it is the most common variation type in humans, thus increasing the compression ratio. The intermediate result is compressed once more, now using the GZIP algorithm. Finally, the compressed genome is stored as output in a single file containing all compressed blocks.

In the decompression phase, JDNA is very similar to FRESCO’s algorithm, but JDNA keeps only a small amount of the reference in the main memory. We slide a vector as the *RP* position pointer advances in the reference, thus occupying a small and fixed quantity of memory. The size of this vector is configurable and, by default, 1MB. The resulting decompressed output is exactly the same as the original to-be-compressed input because JDNA is lossless for files composed of comment lines and A, C, G, T, and N nucleotides. Further details on the implementation of JDNA can be found in S1 Text.

## Results

This section presents the results from a series of experiments focused on measuring the percentage of reference is indexed, the execution time, the compression ratio, and the memory footprint of our solution. We compare the outcomes from JDNA with those from FRESCO, in which we expect to obtain a reduction in execution time, while providing competitive results in terms of compression ratio and memory usage. The experiments analyze broadly accepted variables that should be observed when comparing genome compression tools [9]. Most of our experiments analyze the results separated by chromosomes to isolate the influence of each one of them. Chromosomes with high variability result in a compression ratio naturally lower than that from chromosomes with low variability, which is detailed in Section *Compression Ratio*. Our methodology consists in executing 10 times each experiment with both tools (JDNA and the original FRESCO) and presenting the average value from them. We use a different genome on each execution, which was randomly selected from those 1092 genomes available in the 1000 Genomes Project [20]. The 10 selected genomes are the ones identified by the numbers: HG00173, HG00339, HG00475, HG00619, HG01390, NA12546, NA19449, NA19788, NA20339 and NA20810. We also obtained the reference genome sequence for the human species from the 1000 Genomes Project (human_g1k_v37.fasta).

Our experimental environment is one Dell PowerEdge R410 equipped with two Intel Xeon E5520 processors (2.27GHz), 32GB of RAM memory (1066 MHz), and a hard disk with 146GB (15k RPM). The machine is running the Ubuntu Server 10.04 operating system (64-bit, 2.6.32-21-server kernel). We employed G++ version 4.8.1 for the original FRESCO (https://github.com/hubsw/FRESCO) and Java version 1.7.0 for JDNA. All time measurements were obtained using the time tool from the OS, and the current JDNA implementation is single threaded. The JVM parameters used to run JDNA in this evaluation are presented in S1 Text.

In these tests, we use the following configurable values for JDNA: *K* = 22, *δ* = 120, Δ = 200, block size = 250MB, and decompression window = 1MB. These values were the optimal cases we obtained in our experimental environment.

### Indexing the Reference

**Fig 6:**
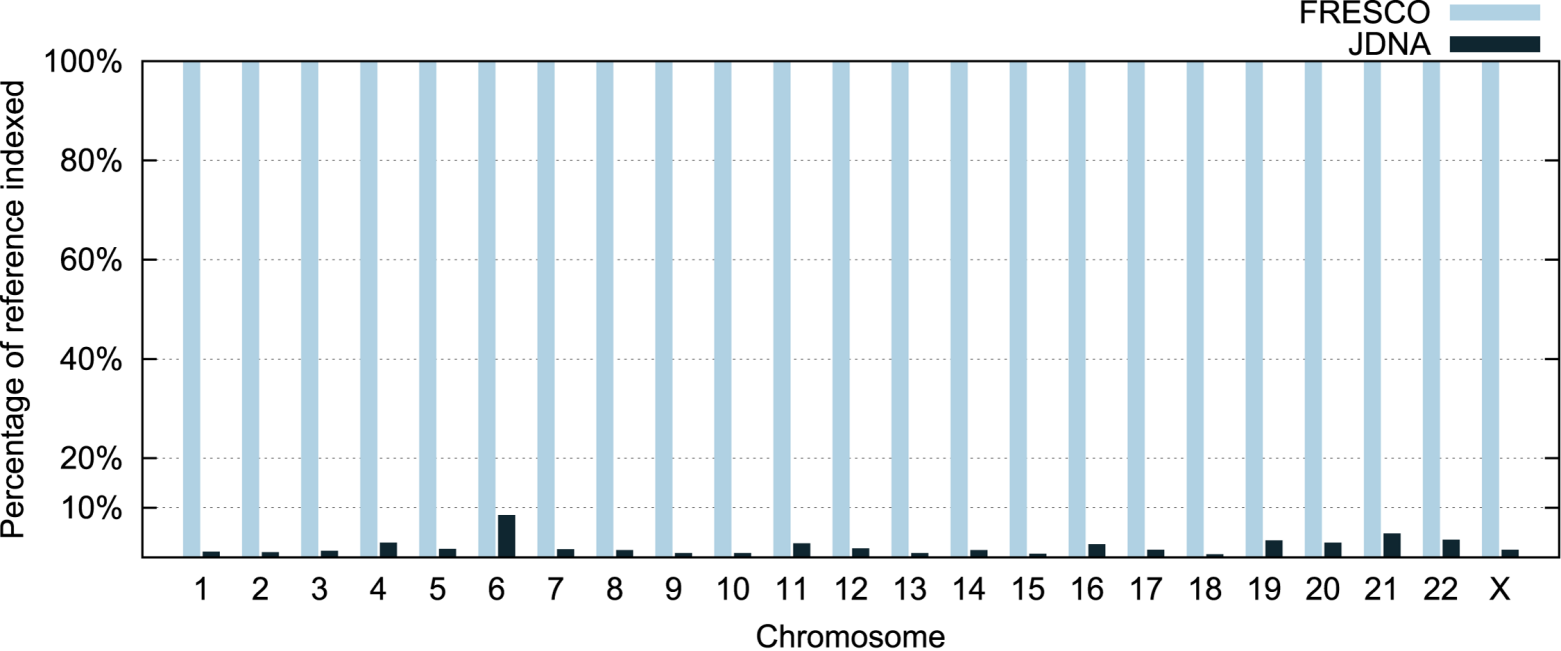
Percentage of the reference indexed by each tool. FRESCO always indexes the full reference (100% for all chromosomes). The percentage of the reference indexed by JDNA varies per chromosome, but is always less than 10%.

Fig 6 presents the percentage of each reference chromosome sequence indexed by each tool. JDNA is able to compress individual genomes indexing less than 10% of each chromosome, where the average percentage is only 2.5%. It reinforces the idea that more than 90% of each chromosome is indexed without a significant contribution to the overall compression process.

### Execution Time

In this experiment, we compare the time efficiency of JDNA and FRESCO for compressing and decompressing human genomes. We separate the evaluation in four components: (C) complete execution, (C1) indexing, (C2) compression, and (D) decompression. They allow us to analyze and understand from which specific execution points the solutions differ.

#### (C) Execution time

**Fig 7:**
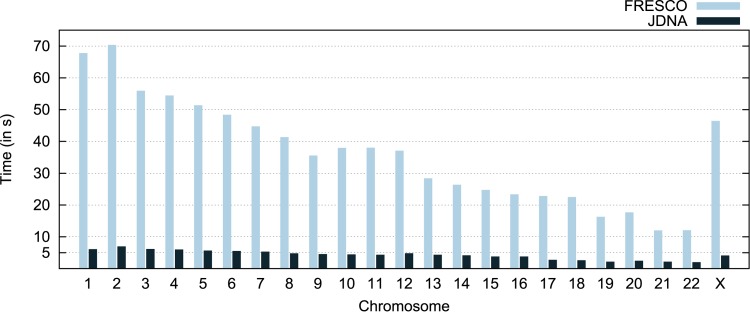
Time spent in the execution of the entire compression workflow. It considers the duration each tool takes to load, index, compress, and write the compressed result to file.

The complete execution of the compression workflow includes the startup time, file reading, memory allocation, reference indexing, compression, encoding the intermediate result and file writing. The resulting values can be seen in Fig 7.

As previously described, JDNA avoids indexing the entire reference genome, which makes a massive difference in the complete compression time. JDNA takes, on average, 4.28 s (seconds) to finish the compression of a chromosome, while FRESCO takes 36.08 s. The results translate to an improvement between 5 to 10 times, meaning a reduction up to an order of magnitude.

#### (C1) Indexing

The time each library spends indexing the reference genome is measured internally during the program execution, and the results can be seen in S1 Table. JDNA spends, on average, only 0.022 s (22 ms) indexing on each chromosome compression, while FRESCO spends 33.9 s. Fig 6 shows the reason for this is the small percentage of base pairs indexed by JDNA, while FRESCO always indexes 100% of the reference genome.

#### (C2) Compression

We measure the time spent effectively compressing the genome and present the results in S1 Table. JDNA takes, on average, 2.28 s per chromosome, while FRESCO takes 0.36 s, where the former is up to 14 times slower than the latter (depending on the chromosome). JDNA spend the majority of its execution time on the compression step. This is expected, since the heuristics have a higher time complexity than a simple table search. However, taking longer in the compression step due to the absence of a full index structure pays off in the full execution time, since there is no indexing step to increase the full execution time. Thus, JDNA’s complete execution time is roughly defined by the time it takes to compress.

#### (D) Decompression

We present in S1 Table the results of measuring the decompression time. JDNA takes, on average, 1.31 s to finish the decompression of a chromosome, while FRESCO takes 1.49 s. These results are expected since the decompression algorithm of both tools is the same.

### Compression Ratio

**Fig 8:**
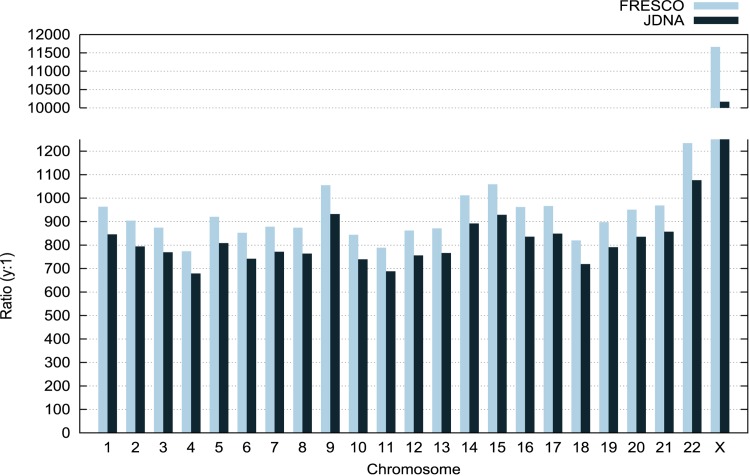
Compression ratio comparison. JDNA’s compression ratio is always close to FRESCO’s ratio, even though always a bit smaller.

A compression ratio of (*y*:*1*) means that the size of a compressed file is *y* times smaller than the size of the original one. Fig 8 compares the achieved compression ratio with each tool. The compression ratios obtained with JDNA and FRESCO differ around 12% on average, and they may happen for two reasons. First, the encoding algorithms used by each tool are different, where a difference of a single bit in the encoding phase amplified by thousands of matches causes a considerable oscillation in the final compressing ratio. Second, FRESCO indexes the entire reference genome, which always finds the best match for each segment, while JDNA indexes only small blocks from the reference. However, in this order of magnitude (compressing a file over 700 ×), the compressed file sizes vary between 6 and 40kB, which we consider a minor difference comparing to the dozens of MB the original file occupies.

**Fig 9:**
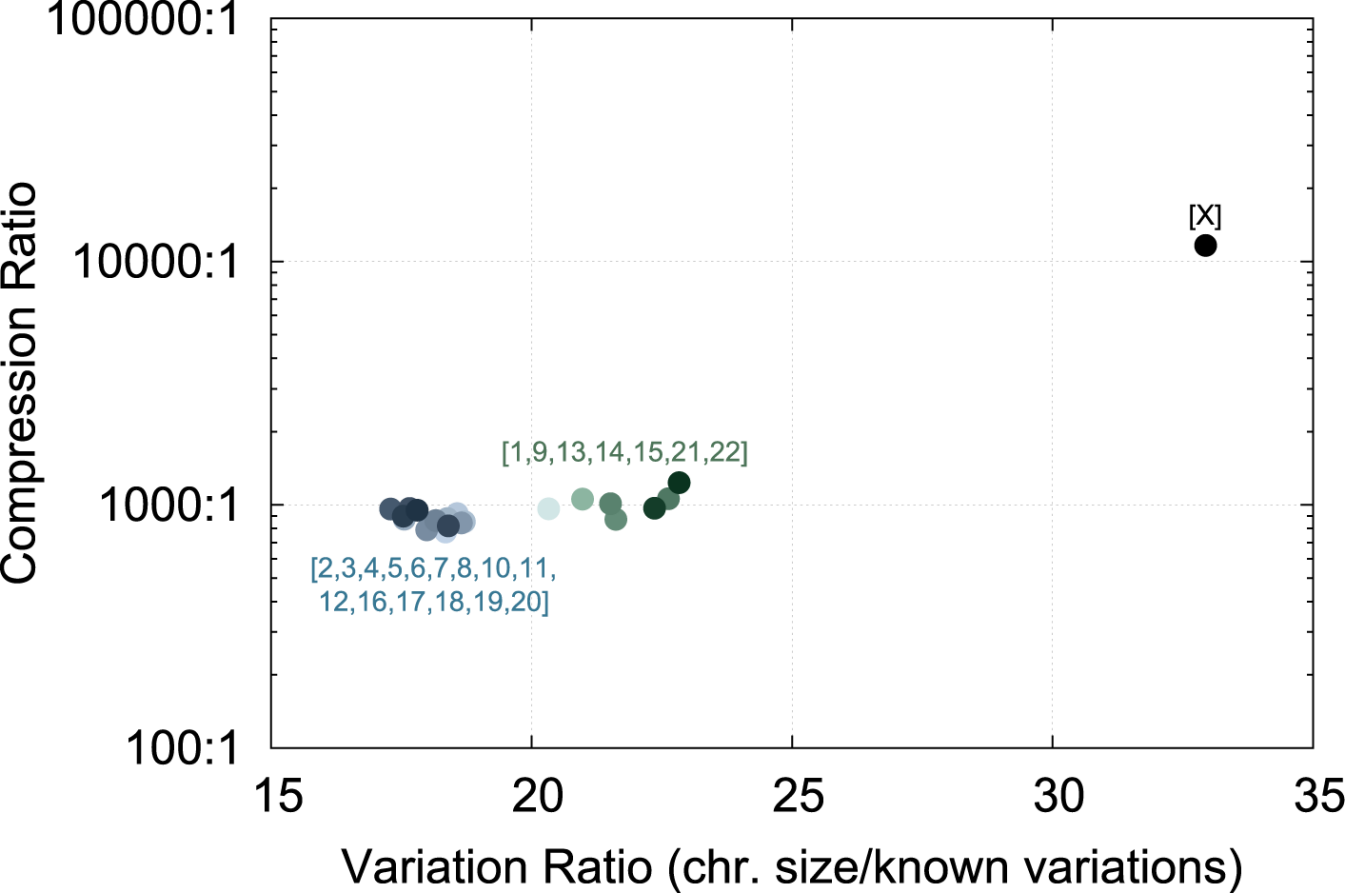
Correlation between the number of variations known by chromosome, and the compression ratio for that chromosome. There are two clear clusters with a similar compression ratio, and the chromosome X has significantly less variations, leading to a higher compression ratio.

Additionally, the compression ratio in chromosome X deviates from the results in other chromosomes. Genomic diversity is smaller in sex chromosomes than in autosomes [21], which explains this outlier in chromosome X. The more genomic variations a block of nucleotides has, the lower compression ratio it results. We obtained the genomic diversity of each chromosome by dividing its approximate size by the number of its known genomic variations available in dbSNP (http://www.ncbi.nlm.nih.gov/snp/, see also the S4 Table). Fig 9 correlates the genomic diversity and the compression ratio of each chromosome, where there are two clusters of points near the variation ratio 20 and the chromosome X isolated near the variation ratio 33. The chromosome X has the least amount of variations [21] per group of nucleotides, which results in the highest compression ratio. On the other hand, for example, yeast genomes can have substantial differences, which results in low compression ratios even from referential compression algorithms [22].

### Memory Footprint

In this experiment, we compare the highest quantity of memory used by each tool during the entire compression and decompression executions. We evaluate the resource requirements of JDNA and FRESCO by measuring their memory peaks, using an external tool, called memusg [23].

#### (C) Compression Memory

In S1 Table, we can observe the memory usage of JDNA and FRESCO during the compression workflow. JDNA has implemented mechanisms that reuse objects and reduce object creation on each instantiation. However, JDNA and FRESCO have similar memory footprints, even after a considerable effort to reduce memory usage of our tool. JDNA and FRESCO’s memory usage differ on average 10%. The bulk of memory usage is due to the way Java creates an integer matrix for the K-mer table in JDNA, where each matrix line is a new object. This incurs in a memory overhead in JDNA, which is the main reason why JDNA uses more memory than FRESCO, even though FRESCO indexes the entire reference.

JDNA was developed in Java, a different programming language than the one used in FRESCO (C++). This choice has consequences since Java requires the initialization of its virtual machine, which also occupies memory space and is included in the values from JDNA in S1 Table. Both tools have three main components that occupy memory: the reference, the to-be-compressed sequence, and the K-mer table. These programs always use at least the main memory space occupied by the sequences, while the remaining used space has the size of the K-mer table as the upper bound. If we only consider the latter, the memory usage would be proportional to the values presented in Fig 6.

#### (D) Decompression Memory

Decompression uses a fixed amount of memory for reference in JDNA (see Section *Implementation Details*), which results in a constant memory usage of about 28MB, as shown in S1 Table. We tested our tool with values of 1, 2, and 5MB (results in S1 Table 2 used a vector size of 1MB). The decompression time was not reduced by increasing the available main memory. Thus, the 1MB window is the most efficient of our test parameters. FRESCO’s memory management on decompression differs from ours, hence the difference in the results.

### Compressing Entire Genomes

We obtain a compression ratio of about 790:1 (∼ 3GB to ∼ 3.6MB) when compressing an entire genome separated by its chromosomes. The compression ratio drops to 750:1 (∼ 3GB to 4.1MB) when compressing the entire genome as a single file. However, these results depend on the similarity between the to-be-compressed and reference genomes, and on how the file blocks are divided.

Compressing the entire genome as a single file takes less time to finish but requires more main memory. It takes about 44 seconds to finish the compression of an entire genome in a single file, while it takes approximately 100 seconds with separated chromosomes. Additionally, the former requires approximately 4GB of main memory to finish the execution.

The reasons for such differences are related to the JVM. Starting only one JVM for the entire genome is more efficient than launching multiple JVMs, one for each chromosome. S1 Text exemplifies how to configure the different JVM parameters.

### De-serialization of the K-mer Table

As stated in the introduction of this article, one may serialize a K-mer table to the disk and de-serialize it at the beginning of a new compression execution. In this experiment, we compare this approach with ODI to verify which is faster when compressing genomes. We completely indexed each chromosome from the reference genome and wrote the resulting K-mer table to a persistent file on the disk. Then, we executed the de-serialization of this data structure 10 times, loading it to the main memory. S1 Table 3 contains the average execution time of de-serialization tests, which were run in Java using parts of JDNA’s code.

These results show that de-serializing a K-mer table spend 25 to 35% less time than creating the table from scratch in FRESCO. The de-serializing approach would reduce the complete execution time of FRESCO for compression, but it still takes approximately 3 times longer than JDNA (see Fig 7 and Table 1). Furthermore, users will have to use storage space to maintain the whole indexed reference on disk. These values attest that avoiding the (de-)serialization is the best approach when compressing single genomes.

**Table 1:** De-serialization averages and standard deviations.

Chr	1	2	3	4	5	6	7	8
Average (s)	14.81	14.46	11.78	11.44	10.83	10.27	9.55	8.79
StDev (%)	0.04	0.14	0.10	0.04	0.03	0.05	0.04	0.03
**Chr**	**9**	**10**	**11**	**12**	**13**	**14**	**15**	**16**
Average (s)	8.51	8.14	8.09	8.07	6.96	6.52	6.22	5.49
StDev (%)	0.14	0.04	0.07	0.06	0.07	0.04	0.06	0.06
**Chr**	**17**	**18**	**19**	**20**	**21**	**22**	**X**	
Average (s)	4.98	4.77	3.67	3.89	3.01	3.19	9.27	
StDev (%)	0.02	0.03	0.01	0.02	0.04	0.02	0.03	

### Compression for Data Transfer

**Fig 10:**
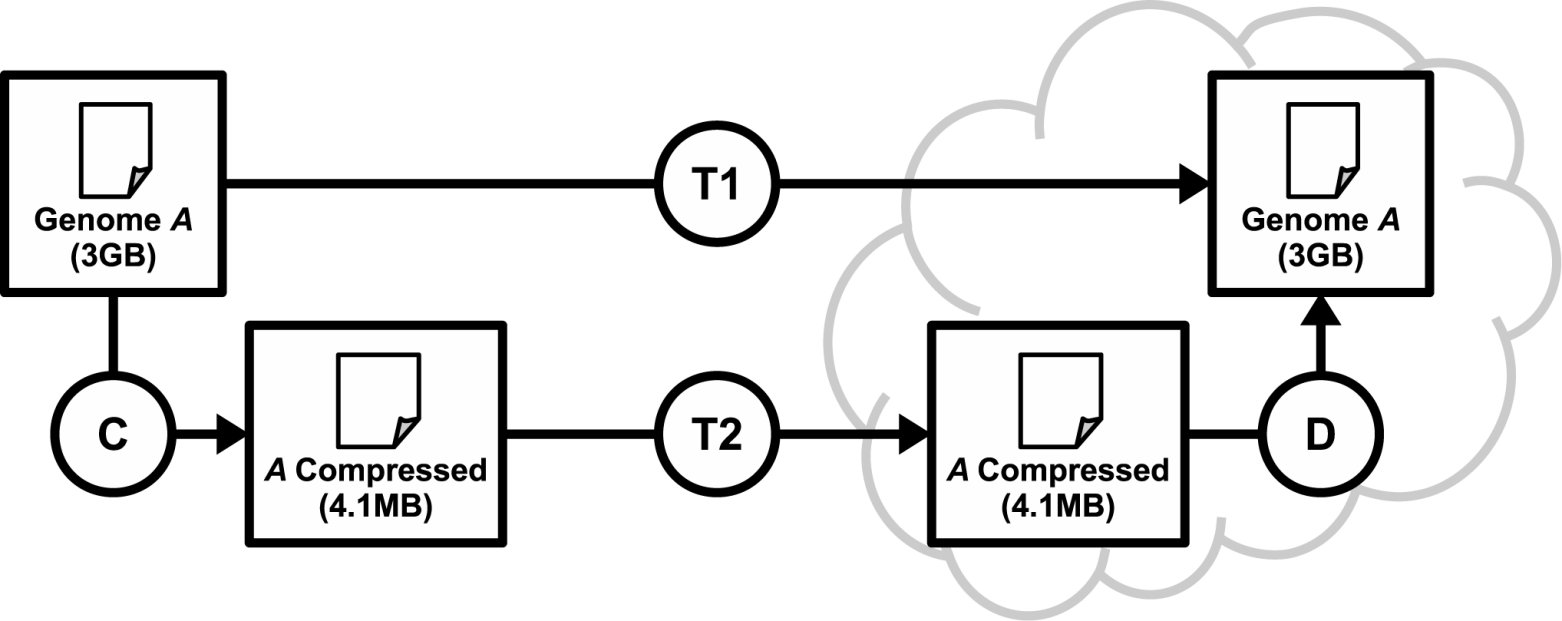
Comparison between the transfer of a compressed and an uncompressed genome. Steps taken to simply transfer a genome file (T1), or to compress (C), transfer (T2) and decompress the file (D).

Compressing files is widely accepted as an efficient solution for storage limitations. However, there are other scenarios where it is advantageous. For example, data transfer may also benefit from compression to reduce the burden of transmitting large files through the network from one point to another. In this experiment, we intend to show that a compress-transfer-decompress workflow is better than a simple transfer workflow when data is as big as a human genome. Fig 10 depicts this scenario, and our experiment considers transfers between our facilities in Portugal and an Amazon EC2 instance (*t2.medium*) running in Ireland. The to-be-transferred genome is the one identified by the accession number HG00173 in the 1000 Genomes Project, which has approximately 3GB uncompressed and 4.1MB compressed. Table 2 presents the average result of each step and its standard deviation.

**Table 2:** Average execution time and standard deviation of the transfer test.

	T1	C	T2	D	Total
Average (s)	285.33	37.96	2.48	38.00	78.45
StDev (%)	2.96	3.79	6.50	7.24	4.93

(T1) corresponds to the simple transfer (no compression), and the Total corresponds to the sum of compression (C), transfer (T2) and decompression (D).

In the first path, the genome is transferred uncompressed (T1 in Fig 10), which takes 285 s (almost 5 minutes) to finish. In the second path, the genome is compressed (C in Fig 10), transferred (T2), and decompressed in the cloud (D). The compression (C) takes 37.9 s to finish, the transfer (T2) takes 2.4 s and the decompression (D) takes 38 s, resulting in a total execution of 78.3 s (less than one minute and half). The latter case is 3.6× faster than the former, which indicates that compressing large genomes is beneficial for both data storage and transfer.

### Conclusions

In this work, we presented the *On-Demand Indexing* (ODI) approach, an optimization for referential compression algorithms applied to DNA sequences. It employs simple heuristics for DNA sequence comparison to avoid indexing the entire reference sequence at the beginning of its execution, an usual process in this type of algorithm. A Java tool, called JDNA, was implemented as a proof-of-concept prototype, and was publicly released as a free and open-source software under the BSD Simplified license in GitHub (https://github.com/Camandros/jdna/).

We evaluated the performance of JDNA and compared it with FRESCO, a state-of-the-art tool for referential compression of genomes. Our results suggest JDNA compresses an entire human genome up to an order of magnitude faster than FRESCO if indexing time is included, while achieving rather similar values for compression ratio, decompression speed, and memory usage. Other tools indexing the entire reference genome may also benefit from ODI approach.

JDNA still has some opportunities for improvement on all properties analyzed in our experimental evaluation. Parallelism may be employed to reduce even more the execution time when compressing several data blocks from an entire human genome. The compression ratio may be increased by employing more efficient encoding algorithms or optimizing up to the limit the implemented ones. The memory usage may also be optimized by improving the configuration of JVM memory management.

## Supporting Information

S1 Text Additional details on JDNA. (PDF)

S1 Table Comparison table between JDNA and FRESCO in the various parameters evaluated in this work. (ODS)

S2 Table Table containing the complete FRESCO results. (ODS)

S3 Table Table containing the complete JDNA results. (ODS)

S4 Table Table containing genomic variation information and its correlation with the compression ratio of JDNA. (ODS)
