# Supplementary material for: On-Demand Indexing for Referential Compression of DNA Sequences
Source: PLoS One. 2015 Jul 6;10(7):e0132460. doi: 10.1371/journal.pone.0132460 (PMC4493149; doi:10.1371/journal.pone.0132460)
Supplement: S1 Text — (PDF) [file pone.0132460.s001.pdf]

On-Demand Indexing for Referential Compression of DNA Sequences

Fernando Alves[1,*,¤] Vinicius Cogo[1] Sebastian Wandelt[2] Ulf Leser[2] Alysson Neves Bessani[1]

1 LaSIGE, University of Lisbon, Lisbon, Portugal
2 WBI, Humboldt-Universität zu Berlin, Berlin, Germany

¤LaSIGE, DI, FC/UL. Room C6.3.35. Campo Grande. 1749 - 016. Lisbon, Portugal.
∗ falves@lasige.di.fc.ul.pt

S1 Text

File Formats. JDNA accepts two file types as input for compression: RAW or FASTA files. If the file is RAW then a CRAW (for compressed RAW) file is generated; if the file is a FASTA then a CRAW and a CCOM files are generated. The CCOM file has the comments present in the FASTA file and the line numbers of those comments. For decompression, a CRAW file is assigned as input. JDNA will then search for a CCOM file with the same name as the CRAW file. If the CCOM file is found then a FASTA file is generated, otherwise, a RAW file is created.

Execution. JDNA is a command line tool, whose arguments are:

```
[TASK] [REFERENCE GENOME] [INDIVIDUAL GENOME] [OUTPUT FILE]
```

where task is either *COMPRESS* or *DECOMPRESS*. An example for compression is:

```
java <JVM parameters> -jar JDNA.jar COMPRESS human_reference.raw \
    HG00173.raw HG00173.craw
```

Java Virtual Machine Configuration. We tune the Java Virtual Machine (JVM) to increase the performance of JDNA [1]. Our tuning aims to avoid garbage collection during execution, and to reduce the re-execution if it is executed. We have no interest on garbage collection execution because most objects created by JDNA on compression are permanent, where even the parallel garbage collection reduces compression speed drastically. If a garbage collection process occurs, then the objects in use are transferred immediately to the permanent memory area of JVM so that in the next garbage collection these objects are not verified again. We tested various JVM configurations, and the following provided the best results for compression tasks:

```
-Xms3000m -Xmx3000m -XX:+ScavengeBeforeFullGC \
    -XX:+UseParallelOldGC -XX:InitiatingHeapOccupancyPercent=80 \
    -XX:InitialTenuringThreshold=20 -XX:NewRatio=1 \
    -XX:SurvivorRatio=1
```

These should be the default options to run JDNA, and the results presented in Section *Results* use them. No parameters are required for decompression. For entire genome compression, one should change `-Xms3000m` to `-Xms4000m` and `-Xmx3000m` to `-Xms4000m` and maintain the other parameters. For debugging purposes, one may add:

```
-XX:+PrintGCDetails -XX:+PrintGCTimeStamps -verbose:gc
```

References

1. Hunt C, John B. Java Performance. 1st ed. Upper Saddle River, NJ, USA: Prentice Hall Press; 2011.
